# FREQUENCY RESPONSE AND DISTORTION PROPERTIES OF RECONSTRUCTION ALGORITHMS IN COMPUTED TOMOGRAPHY

**DOI:** 10.1093/rpd/ncab058

**Published:** 2021-05-06

**Authors:** Joel Larsson, Magnus Båth, Anne Thilander-Klang

**Affiliations:** Department of Radiation Physics, Institute of Clinical Sciences, Sahlgrenska Academy, University of Gothenburg, Gothenburg SE-413 45, Sweden; Section of Diagnostic Imaging and Functional Medicine, NU Hospital Group, Trollhättan SE-461 85, Sweden; Department of Radiation Physics, Institute of Clinical Sciences, Sahlgrenska Academy, University of Gothenburg, Gothenburg SE-413 45, Sweden; Department of Medical Physics and Biomedical Engineering, Sahlgrenska University Hospital, Gothenburg SE-413 45, Sweden; Department of Radiation Physics, Institute of Clinical Sciences, Sahlgrenska Academy, University of Gothenburg, Gothenburg SE-413 45, Sweden; Department of Medical Physics and Biomedical Engineering, Sahlgrenska University Hospital, Gothenburg SE-413 45, Sweden

## Abstract

Denoising reconstruction techniques can introduce nonlinear properties into computed tomography (CT) systems. These nonlinear algorithms introduce distortion which affects the assessment of the resolution of the system. The purpose of the present study was to decouple and investigate amplitude modulation and waveform distortion in reconstruction algorithms in CT. The methodology developed by Wells, J. R. and Dobbins, J. T. III [*Frequency response and distortion properties of nonlinear image processing algorithms and the importance of imaging context*. Med. Phys. 40, 091906 (2013)] was adapted to CT reconstruction algorithms. The CT simulating program ASTRA Toolbox© for MATLAB™ was used for the reconstruction of the sinusoidal wave functions. Filtered back projection and the simultaneous iterative reconstruction technique were investigated with simple nonlinear mechanisms: a median filter and a non-negative constraint, respectively. The native reconstruction algorithms were not free from nonlinear waveform distortion, however, none of the metrics showed any dependence on the contrast-to-noise ratio (CNR). Furthermore, the algorithms including nonlinear mechanisms showed a clear and specific CNR dependence, indicating the necessity for distortion analysis in nonlinear CT reconstruction.

## INTRODUCTION

Nonlinear denoising reconstruction algorithms have been used in computed tomography (CT) for more than 10 y to produce images with less noise and/or to reduce the absorbed dose to the patient^(1,2)^. It is still not possible to completely characterise the effect of denoising in the image analysis of such algorithms. New task-based metrics^(3–7)^ describing the resolution properties of nonlinear CT iterative reconstruction techniques have been shown to be useful in approximating the resolution performance, and the resolution has been found to depend on the contrast and noise magnitude^(3)^. However, the effects of distortion arising from nonlinear denoising reconstruction algorithms have not been taken into account in these metrics, and these could affect the assessment of the resolution of the system.

A better understanding and characterisation of the nonlinearities in denoising algorithms are therefore necessary to help identify which elements in the algorithms cause distortion in the resulting clinical images. Wells and Dobbins^(8)^ have taken steps in this direction and have developed metrics describing the often-ignored influence of signal distortion resulting from nonlinear algorithms. They have been able to decouple signal amplitude modulation from nonlinear waveform distortion. The 1D method of analysing nonlinear systems, the total harmonic distortion (well-known in engineering communities), has been modified and implemented in 2D systems such as X-ray images. This modification resulted in two metrics referred to as the principle frequency response (PFR) and the distortion power spectrum (DPS) and two figures of merit (FOMs): the distortion index (DI) and sum of DI (ΣDI). These modified metrics and FOMs have shown correlated noise and backgrounds of anatomical structures to have an effect on nonlinear resolution, indicating that nonlinear distortion should be evaluated at relevant backgrounds.

In practice, a CT system can be approximated as linear. However, strictly speaking, a CT scanner will suffer from nonlinearities due to physical limitations such as discontinuities in the projection acquisition and sampling distance. In addition to aliasing, distortion arising from these nonlinearities was observed as streaking artefacts in the early days of CT and was reduced by improvements in geometry, e.g. the number of detector elements, projection angles^(9,10)^, extended CT scale technique^(11,12)^ and by convolution kernels^(13)^. However, the reconstruction of highly attenuating materials, such as metal implants, will still suffer from some visual distortion originating from the CT geometry^(14)^. These inherent nonlinearities will always be present in the distortion analysis of CT systems. Reconstruction by filtered back projection (FBP) does not add any new nonlinearities, as this reconstruction method is linear, and the inherent distortion effects are simply weighted differently when using different convolution kernels. Knowledge of the distortion properties of CT systems can be used to understand and reveal the properties of nonlinear reconstruction algorithms. This study focuses on implementing the above-mentioned metrics of frequency response and distortion properties in CT in order to demonstrate their ability to quantify the distortion associated with nonlinear reconstruction algorithms.

## MATERIALS AND METHODS

The metrics PFR and DPS and the FOMs DI and ΣDI have been described by Wells and Dobbins for 2D systems^(8)^. These quantitative measures characterise the nonlinearity of a system by decoupling the signal amplitude modulation from nonlinear distortion effects. In practice, a signal superimposed on a noisy background is processed with a noise reduction filter and is then resolved by subtracting exactly the same background, processed without the signal present. This could be done with an arbitrary signal and background. However, sinusoidal wave functions are well established in Fourier-based analysis, and the response amplitude modulation in the Fourier domain can be easily distinguished from the nonlinear distortion effects.

### Sinusoidal response

2D narrow-band sinusoidal wave functions were used as task objects in the analysis of distortion properties. These have been investigated elsewhere^(8)^ and can be expressed as:

(1) \begin{equation*} {s}_{u_0,{v}_{0,}{\varphi}_0}\left(x,y\right)=A\cos \left[2\pi \left({u}_0x+{v}_0{y}+{\varphi}_0\right)\right] \end{equation*}

where the sinusoids, *s*_*u*0*,v*0*,φ*0_(*x*,*y*), can be modified by the amplitude, *A*, the spatial frequencies in the Cartesian coordinates, *u*_0_ and *v*_0_, and their respective spatial locations, *x* and *y*, and the phase, *φ*_0_. In this study, the sinusoids were windowed with a circular mask and then forward-projected $\mathcal{P}$(*s*_*u*0*,v*0*,φ*0_(*x*,*y*)) into sinograms, and a background of Poisson noise, *b*(*p*,*q*), was superimposed depending on the sinogram, where *p* and *q* are the number of projection angles and detector elements, respectively. A signal sinogram with added background noise reconstructed with an arbitrary algorithm *f* from which the same background noise was subtracted and reconstructed separately with the same algorithm *f* can be written as follows:

(2) \begin{eqnarray*} {t}_{u0,v0,{\varphi}0,b}(x,y)&=&f(\mathcal{P}({s}_{u_0,{v}_{0,}{\varphi}_0})(p,q)+b(p,q))\nonumber\\&&\left(x,y\right)-f\left(b\left(p,q\right)\right)\left(x,y\right) \end{eqnarray*}

The output image, ${t}_{u0,v0,{\varphi}0,b}(x,y)$, is the result of processing *s*_*u*0*,v*0,*φ*0_ in the context of a Poisson noise background, and both the principle frequency modulation and harmonic distortion of the sinusoid response were isolated by subtracting *f*(*b*(*p*,*q*))(*x*,*y*).

**Figure 1:**
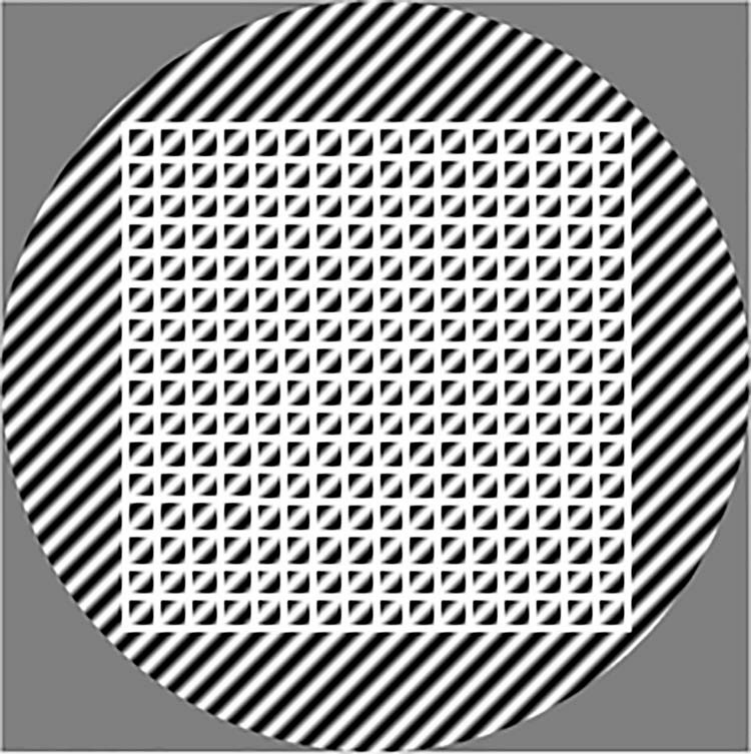
example of an original sinusoidal task object (before forward projection) masked circularly to reduce distortion due to the reconstruction of square objects; the sinusoid has a matrix size of 768 × 768 pixels and horizontal and vertical frequencies *u_0_* = 0.03125 pixel^−1^ and *v_0_* = 0.03125 pixel^−1^, respectively; the borders of the white mesh (512 × 512 pixels) indicate the area of one task object, and the mesh (consisting of 16 × 16 squares) illustrates the minimum number of repeated sinusoids in one task object, i.e. 256 repetitions.

Sinusoidal task objects for which a whole number of periods fitted in a 32 × 32 pixel matrix were repeated to form a 768 × 768 pixel matrix before reconstruction. The analysis of the transferred distortion power was performed by using a 512 × 512 pixel region of interest (ROI) (see Figure 1). Thus, the sinusoidal task objects were multiplied by the finite spatial domain of the ROI (a box function). The spatial domain multiplication of a sinusoid and a box function is in the Fourier domain represented by a convolution of two delta functions and a 2D sinc function. However, calculating the discrete Fourier transform (DFT) of a sinusoid for which a whole number of periods fits into the ROI corresponds to sampling the resulting sinc functions at their zero-value valleys. Hence, the DFT of these narrow-band sinusoids have the appearance of a sampled infinite sinusoid. Thus, distortion originating from the truncation of the sinusoids was restricted to the actual reconstruction dimensions of a typical CT image. The size of the ROI was chosen to repeat the sinusoidal task object at different backgrounds at least 256 times and to fit within the reconstructed volume, excluding possible edge distortion effects (see Figure 1). Furthermore, the phase dependence of a task object was accounted for by evenly distributing the phase shifts of each spatial frequency and by excluding phase shifts resulting in null values, resulting in a total number of 30 phase shifts.

Reconstructing square objects with circular reconstruction algorithms may lead to distortion affecting the distortion analysis. A circular mask was therefore applied to the simulated task objects in an attempt to minimise this distortion. The pixel diameter of the mask was constrained to the number of pixels in the reconstruction volume (768 pixels) (Figure 1).

### PFR, DPS, DI, ΣDI and $\overline{\mathrm{DI}}$

The waveform response of a sinusoid processed by a nonlinear system was quantitatively measured by the PFR and the DPS, decoupling the amplitude modulation from nonlinear distortion effects. PFR provides a measure of how the amplitude of the original task object (sinusoid) is modulated by a nonlinear algorithm and can be written as follows^(8)^:

(3) \begin{equation*} \mathrm{PFR}\left(\mathrm{u},\mathrm{v}\right)=\underset{M,N\to \infty }{\lim}\frac{1}{MN}\sum_{m=1}^M\left|\frac{F_{2\mathrm{D}}\left({\sum}_{n=1}^N{t}_{u,v,{\varphi}_m,{b}_n}\right)\left(u,v\right)}{F_{2{\rm D}}\left({s}_{u,v,{\varphi}_m}\right)\left(u,v\right)}\right| \end{equation*}

The variables *M* and *N* denote the number of phase shifts, *φ_m_*, and background samples, *b_n_*, respectively, and *F*_2D_ denotes the 2D DFT. In this study, *N* = 1, however, each sinusoidal task object was repeated at least 256 times in that background (Figure 1). The fraction of signal power preserved to the principle frequency after the processing of a nonlinear algorithm is equivalent to the square of PFR^(8)^. DPS describes the power transferred to all other frequencies except the principle frequency due to nonlinear distortion. DPS provides an independent measure of the amount of signal power distributed from a principle spatial frequency to all nonprinciple frequency components^(8)^. This is achieved by using a notch filter (Σ_(*u*_*i*_,*v*_*j*_) ≠ ±(*u*,*v*)_) that ignores any changes at the principle frequency component and can be written as follows^(8)^:

(4) \begin{eqnarray*} \mathrm{DPS}\left(u,v\right)=\underset{N_x,{N}_y\to \infty }{\lim}\underset{M,N\to \infty }{\lim}\frac{N_x{N}_y\Delta x\Delta y}{M}{\sum}_{m=1}^M\nonumber\\{\sum}_{\left({u}_i,{v}_j\right)\ne \pm \left(u,v\right)}{\left|\frac{F_{2\mathrm{D}}\left({\sum}_{n=1}^N{t}_{u,v,{\varphi}_m,{b}_n}\right)\left({u}_i,{v}_j\right)}{N}\right|}^2 \end{eqnarray*}

In the analysis, variables *N_x_* and *N_y_* are the ROI array dimensions (*N_x_* = *N_y_* = 512, in this study), and *∆x* and *∆y* are the pixel dimensions (*∆x* = 1 and *∆y* = 1, in this study) and *F*_2D_ denotes the 2D DFT. These variables convert the output units so as to reflect the signal power. The amplitude of a sinusoidal task object transferred through a linear system will be modulated exclusively and will generate no dispersion to other spatial frequencies. Hence, for linear systems, PFR would be reduced to the system modulation transfer function (MTF), and DPS would be zero at all spatial frequencies. Theoretically, according to the formalism of PFR and DPS, the variables *M*, *N*, *N_x_* and *N_y_* should go to infinity or be large enough to represent a system processing an infinite signal. However, this is never the case for a CT system. *N_x_* and *N_y_* were bound to the limits of the investigated CT system, and *M* and *N* were assumed to be large enough, as the result did not alter between repeated calculations with resampled noise or an increased number of phase shifts.

DI(*u*,*v*) describes the nonlinear distortion power as a fraction of the total output signal power at each principle frequency^(8)^:

(5) \begin{equation*} \mathrm{DI}\left(u,v\right)=\frac{\mathrm{DPS}\left(u,v\right)}{\ {N}_x{N}_y\mathit{\Delta }x\mathit{\Delta }y{\left|A/2\mathrm{PFR}\left(u,v\right)\right|}^2+\mathrm{DPS}\left(u,v\right)\ } \end{equation*}

DPS was normalized to the total output signal power expressed as the sum of the power of the principle frequency (where *A* is still the amplitude) and nonlinear distortion. The division by 2 is used to compensate for the signal power of the paired Fourier domain components. A scalar measure of DI was expressed in the convenient FOM ΣDI^(8)^:

ΣDI$=\frac{\sum \sum \mathrm{DPS}(u,v)}{\sum \sum [{N}_x{N}_y\mathit{\Delta }x\mathit{\Delta }y{|A/2\mathrm{PFR}(u,v)|}^2+\mathrm{DPS}(u,v)]}$(6)

where the summations are over the investigated principle frequencies, *u* and *v*, and the computation quantifies the fraction of the total output signal power attributed to the nonlinear waveform distortion. However, it could also be of interest to consider the mean nonlinear waveform DI:

(7) \begin{equation*} \overline{\mathrm{DI}}=\frac{\sum \sum \mathrm{DI}\left(u,v\right)}{N_{u,v}} \end{equation*}

where the summations are over the investigated principle frequencies, *u* and *v*, and *N*_*u*,*v*_ is the total number of principle frequencies used in the analysis of DI(*u*,*v*). This fraction determines the mean distortion of all individual frequencies of a nonlinear system. In contrast to ΣDI, the mean value of DI(*u*,*v*) ($\overline{\mathrm{DI}}$*)*is less sensitive to the variations in the output signal power of the system, as the fraction of each principle frequency would contribute equally to the FOM.

### Simulation of CT reconstruction

Reconstruction was performed using the open source Compute Unified Device Architecture (CUDA)-integrated CT simulation toolbox ASTRA©^(15–17)^ for MATLAB™. The geometry of the reconstructed volume was specified so as to generate quadratic images of the size 768 × 768 pixel on an area of 768 × 768 mm^(2)^. In this study, the volume geometry constrained the theoretical maximum resolution to 0.5 cycles per mm. The projection geometry of the reconstruction was that incorporated in the toolbox, i.e. fan-beam using a flat array of detectors (an arc array of detectors had not been incorporated into the toolbox at the time of the present study) with 737 detector elements and 1.2858 mm detector element width defined at the iso-centre, meaning that it is possible to receive a maximum spatial frequency of 1/2.5716 mm^−1^. A total of 1152 projection angles were evenly spaced around 360°, a source-to-iso-centre distance of 696.7 mm and a source-to-detector distance of 1085.6 mm. Sinograms of the generated sinusoids were computed through forward projection using a function incorporated in the toolbox accounting for volume and projection geometry.

### Nonlinear reconstruction

Theoretically, the reconstruction of a CT image involves solving a linear inverse problem. The reconstruction algorithm FBP and the native version of other iterative reconstruction techniques, e.g. the simultaneous iterative reconstruction technique (SIRT) and Krylov subspace methods (e.g. conjugate gradient least squares), solve the problem using a linear method. In practice, the CT geometry will introduce nonlinearities independently of the reconstruction algorithm. Hence, the finite number of projections and pathways of a CT system will make it difficult to reproduce sharp edges without discontinuities, and the linear approximation of a CT system will not always be valid since the criteria of shift-invariance and stationarity may not be fulfilled^(18)^. Historically, this has been circumvented, for example, in FBP reconstruction, by applying smoothing kernels to supress the resulting nonlinear distortion (e.g. streaking artefacts), and such a system can often be approximated as linear. However, applying image processing algorithms or additional constraints to the reconstruction chain could add nonlinearities, invalidating the linear approximation.

Two reconstruction algorithms were investigated in this study: FBP (using a Ram-Lak ramp filter) and SIRT. A 3 × 3 median filter was applied to the sinogram before reconstruction to introduce nonlinearity in the reconstruction chain of FBP (FBP_median_), while a non-negative constraint was incorporated into the SIRT algorithm (SIRT_non-negative_). The SIRT algorithm was initialised with an image reconstructed with FBP and was terminated after 100 iterations.

### Evaluated contrast-to-noise ratios

**Figure 2:**
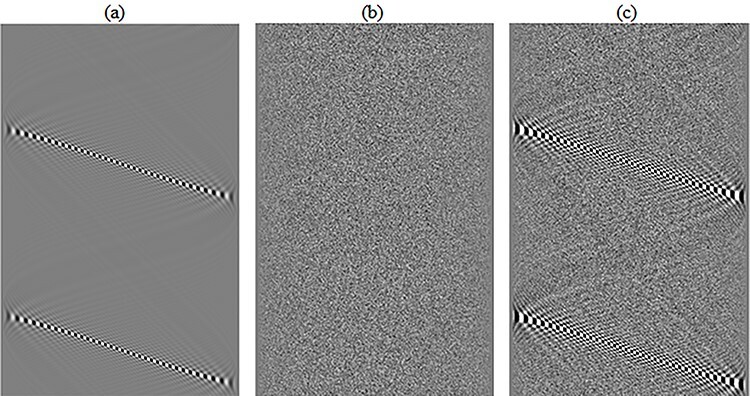
sinograms of (**a**) a circularly masked original sinusoid, (**b**) a Poisson noise map originating from the sinogram of the original sinusoid (with contrast between that of water and air) and (**c**) the Poisson noise map added to the sinogram of the original sinusoid.

Background noise texture has been shown to influence the performance of nonlinear image processing algorithms^(8)^. However, the present study was focused on the implementation of frequency response and the distortion properties of reconstruction algorithms in CT. Therefore, the analysis was simplified and focused on the properties of Poisson noise added directly to the sinogram (Figure 2c). The Poisson noise maps (Figure 2b) were simulated to originate from the projections of the sinusoidal task objects (Figure 2a) with contrast between that of water and air:

(8) \begin{eqnarray*} {\mathrm{Noise}}_{\mathrm{map}}\!\!\!\!\!\!&=&\!\!\!\!\!\!\mathrm{Poisson}\left({\lambda} \left[1-{e}^{-{\rm L}_{\mathrm{norm}} \left({\mu}_{\mathrm{water}}-{\mu}_{\mathrm{air}}\right)}\right]\right)\nonumber\\&&\!\!\!\!\!\!-\lambda \left[1-{e}^{-{\rm L}_{\mathrm{norm}} \left({\mu}_{\mathrm{water}}-{\mu}_{\mathrm{air}}\right)}\right] \end{eqnarray*}

where the noise function generated random numbers from a Poisson distribution with the expected value ${\lambda}$. The second term subtracted the sinogram of the sinusoid to generate only the noise variation. The factor 1 − *e*^−(…)^ describes the number of photons reaching the detector elements, where L_norm_ is a normalized sinogram of the sinusoidal task objects ranging from 1 to 2, and *μ*_water_ and *μ*_air_ are the linear attenuation coefficients of water (liquid) and air (dry, near sea level), respectively. The coefficients were calculated using the mass attenuation coefficients at 60 keV taken from the NIST database^(19)^, representing, approximately, the mean photon energy of a CT examination at a tube voltage of 120 kV. The difference between *μ*_water_ and *μ*_air_ was weighting the L_norm_ to describe the sinusoidal task objects in terms of water and air. The contrast was defined as the greatest variation from the mean intensity of the sinusoids. The contrast-to-noise ratio (CNR) of the task objects was obtained by keeping the amplitude, $A$, of all the sinusoids with a contrast between that of water and air constant and by allowing ${\lambda}$ in the Poisson noise calculation to vary. CNR values of 0.0625, 0.125, 0.25, 0.5, 1, 2, 4 and 8 of the sinusoidal task objects were investigated to determine the changes in the performance of the CT reconstruction algorithms. The CNR was defined in a 512 × 512 pixel ROI in an FBP-reconstructed image of a task object at horizontal and vertical frequencies of *u*_0_ = 0.03125 mm^−1^, *v*_0_ = 0.03125 mm^−1^, respectively.

## RESULTS

### Principle frequency response

**Figure 3:**
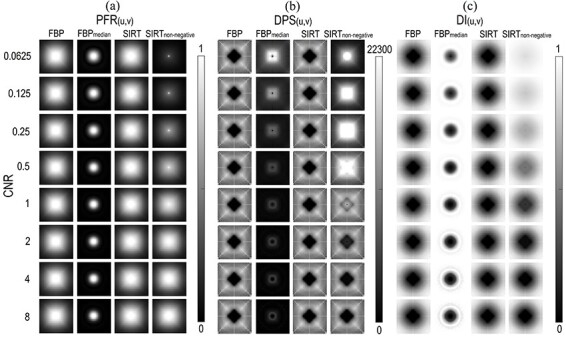
(**a**) PFR(*u*,*v*), (**b**) DPS(*u*,*v*) and (**c**) DI(*u*,*v*) at CNRs ranging from 0.0625 to 8; the native FBP and SIRT algorithms (initialised with an FBP image) exhibited no CNR dependence; the addition of a nonlinear mechanism to each algorithm (a median filter for FBP and a non-negative constraint for SIRT) introduced a CNR dependence unique to each mechanism; the images (32 × 32 pixel) corresponding to each metric are equally scaled; the principle frequency representing the DC component is positioned at the centre of the metric image, and the sampled principle frequencies increase circularly outwards from this point; the intensity scale of the DPS(*u*,*v*) images extends from 0 up to 22 300 and cuts off the lowest frequencies of SIRT with a non-negative constraint at CNR below 1 to emphasise the differences between the other algorithms; the image intensity of DPS(*u*,*v*) has the units of pixel value^2^ × pixel area, the other metrics are unitless.

**Figure 4:**
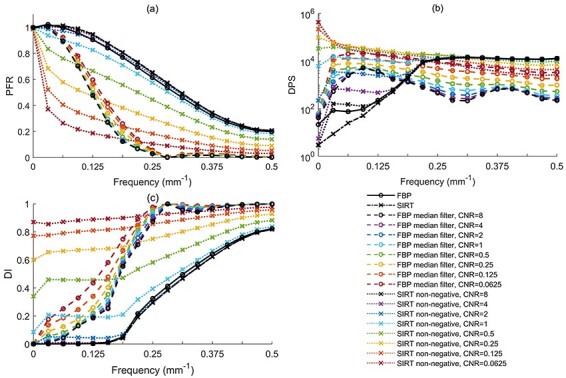
horizontal (0°) profiles of (**a**) PFR(*u*,*v*), (**b**) DPS(*u*,*v*) and (**c**) DI(*u*,*v*) are shown to allow the comparison of the amplitudes of the various algorithms for different values of CNR; it should be noted that the native FBP and SIRT algorithms overlap at all values of CNR, and a single line is shown in black for each of these algorithms; it should also be noted that DPS(*u*,*v*) is plotted on a log scale

The PFR without the addition of nonlinear components was independent of the CNR, and both the FBP and SIRT algorithms had approximately the same Gaussian shape (Figure 3a). However, the PFR of the SIRT algorithm was slightly better than that of the FBP algorithm at almost all the principle frequencies investigated (as can be seen from the horizontal PFR profile (0°) in Figure 4a). The applied median kernel showed a distinct difference in the PFR pattern compared to the other algorithms. The PFR of FBP_median_ had a narrow sinc-like shape at all CNRs and increased slightly with decreasing CNR. However, the frequency response of FBP_median_ above ~0.25 mm^−1^ was the lowest among all the algorithms and CNRs investigated. The shape of the PFR when using the SIRT_non-negative_ algorithm was the same as that without the constraint at high CNR. The PFR of this algorithm decreased substantially at frequencies above the zero frequency (direct current, DC) component when the CNR decreased below 2 (Figures 3a and 4a).

### Distortion power spectrum

The DPS had roughly the same order of magnitude for all the algorithms for values of the CNR above 1 (Figures 3b and 4b). For CNR below 1, the distortion of the DC component given by the SIRT_non-negative_ algorithm was ~50 times higher than for the other algorithms. However, the DPS given by the SIRT_non-negative_ algorithm at the higher principle frequencies investigated decreased with decreasing CNR (see Figure 3b) and showed a lower distortion power than the native SIRT algorithm (the PFR for SIRT_non-negative_ was also lower in this frequency range).

The distortion power of the FBP and SIRT algorithms without the nonlinear components remained constant for all values of CNR and had approximately the same shape (Figure 3b). Furthermore, the native SIRT algorithm had an overall slightly lower DPS than the FBP (Figure 4b). The DPS response function of the FBP_median_ algorithm implied a general increase in the nonlinear distortion with decreasing CNR. This increase in the distortion power of the FBP_median_ algorithm was more prominent in the mid-frequency range than in the high range (Figure 4b).

### Distortion index

DI showed that the fraction of the output signal power attributed to the nonlinear distortion at all principle frequencies investigated dominated increasingly as the CNR was reduced, when applying the nonlinear components to both algorithms (median filter and non-negative constraint). This was most prominent for SIRT_non-negative_ (Figures 3c and 4c). The PFR component dominated as an inverted narrow sinc-shaped function in the DI response of the FBP_median_ algorithm (compare Figure 4a and c). Furthermore, the DI increased with decreasing CNR for both the algorithms, including nonlinear components. The native SIRT algorithm had the lowest DI at almost all principle frequencies investigated; the small difference being most prominent in the mid-frequency range (see Figure 4c).

### ΣDI and $\overline{\mathrm{DI}}$

**Figure 5:**
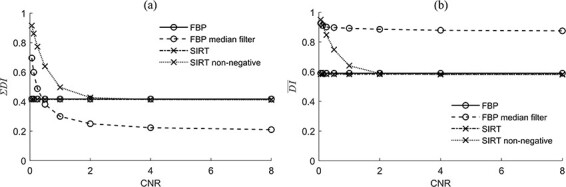
(**a**) ΣDI and (**b**) $\overline{\mathrm{DI}}$ as a function of CNR showing the overall impact of distortion; these plots show the impact of adding the nonlinear mechanisms to the FBP and SIRT algorithms (median filter and non-negative constraint, respectively); it can be seen that the native algorithms show no dependence on CNR in contrast to the nonlinear mechanisms, which show a clear dependence; $\overline{\mathrm{DI}}$ is less sensitive to the differences in total output and may be used as a complement to ΣDI.

Neither the FBP nor the SIRT algorithm showed any CNR dependence in the range investigated (see plots of ΣDI and $\overline{\mathrm{DI}}$ in Figure 5). ΣDI and $\overline{\mathrm{DI}}$ showed increased distortion when the CNR decreased for both the FBP_median_ and the SIRT_non-negative_ algorithms. At high values of CNR, $\overline{\mathrm{DI}}$ for the FBP, SIRT and SIRT_non-negative_ differed from ΣDI by a factor of 1.4, and for the FBP_median_ algorithm, by a factor of 4 (Figure 5a and b). The value of ΣDI and $\overline{\mathrm{DI}}$ for the SIRT_non-negative_ algorithm tended towards that of the native SIRT algorithm, and the difference was reduced at CNR above 2. The value of ΣDI for FBP_median_ had the lowest signal power attributed to nonlinear distortion as a fraction of the total output signal power at CNR above 0.5 (Figure 5a). FBP_median_ also generated approximately one order of magnitude lower total output signal than the other algorithms. $\overline{\mathrm{DI}}\overline{}$ was less sensitive to the total output signal and reflected the mean DI over the principle frequencies investigated. The highest mean distortion among the algorithms investigated was seen for FBP_median_ at values of CNR above 0.125 (Figure 5b). The lowest value of $\overline{\mathrm{DI}}$ was obtained with SIRT at all values of CNR.

## DISCUSSION

Nonlinear image denoising has been an expanding area in medical CT imaging for more than a decade. The initiation was a computer capacity large enough to manage comparable reconstruction times with incorporated iterative mechanisms to that of FBP. A nonlinear denoising mechanism which is frequently used is a type of regularisation technique that is based on the minimization of the total variation. This prior object information modelling has the ability to reduce noise while preserving sharp edges by minimising the derivative of the image data^(1)^. Another iterative approach with Bayesian statistical noise models and geometrical modelling has also been used in denoising algorithms to address the problem of noise reduction^(20)^. These algorithms have been found to smoothen the noise and improve the spatial resolution in clinical trials^(21–24)^ and phantom studies^(25)^. The traditional general metric for spatial resolution, the MTF, has been modified to be task-specific and incorporated in a detectability index *d*′^(26,27)^. This modification takes the dependence on CNR into consideration, however, it ignores the properties of the distortion. Distortion has traditionally been managed using primarily linear reconstruction filters to reduce streaking artefacts originating from the geometrical configuration of CT systems, e.g. the inevitable discretisation of the detector elements^(14)^. Furthermore, the need for metrics in distortion analysis was reduced, as thinner detector elements were developed, and more detectors were used in the detector arc. However, the transition from FBP to nonlinear image denoising algorithms has highlighted the need for metrics that analyse the properties of distortion. In this study, the metrics PFR, DPS, DI and ΣDI, originally developed and applied to conventional radiography^(8)^, were implemented in CT through simulation to illustrate the concept and its ability to assess the distortion of the image quality arising from the nonlinear CT reconstruction algorithms. Two different nonlinear mechanisms were investigated in the present study: an applied median filter on the FBP algorithm and a non-negative constraint on the SIRT algorithm in order to identify the basic properties of filter kernels and intensity constraints. However, knowledge is required on other reconstruction mechanisms for a complete analysis of the distortion properties of modern noise reduction algorithms.

The metrics used in this study showed that the native analytical reconstruction algorithm FBP and the native iterative reconstruction algorithm SIRT did not vary over a wide range of CNR values and only expressed the inherent nonlinear distortion arising from the geometry of the CT system. The slight improvements in PFR and DPS for the SIRT algorithm, compared to FBP, were expected (Figure 4). The improvements were also expressed by ΣDI and $\overline{\mathrm{DI}}$, although this can barely be seen in Figure 5. An FBP image was used as the starting image in the iteration chain of the SIRT algorithm, and the pixels were updated to better fit the acquired projection data. The FBP algorithm was investigated with the Ram-Lak filter to specifically illustrate the inherent distortion in a CT system. This inherent distortion could thus be analysed in relation to the distortion originating from the use of nonlinear reconstruction algorithms.

The median filter applied to the sinogram introduced aliasing in the frequency domain depending on the size of the filter kernel. One result of this truncation was seen in the PFR and DI plots as a sinc function. The shape of the sinc function may reflect the 3 × 3 kernel of the median filter, where the spatial distance to the next pixel was bound to the detector size (horizontally) and the projection angle shift (vertically). These distances will determine the resolution degradation, as convolution with a large spatial box-function (filter kernel) is analogous to a narrow sinc function in the frequency domain. The present detector configuration represents a rather old CT system. The degradation in resolution due to the size of the filter kernel may be smaller in modern CT systems, where the distance between the detector elements is smaller and the number of projections in one revolution is higher. However, the present configuration was used to emphasise the aliasing effect to enable the dependence on CNR to be seen more easily.

The increase in PFR seen with the FBP_median_ algorithm when the CNR decreases may seem unintuitive. However, the median filter may have distorted the frequency power of the noise to give a contribution to the power of the analysed principle frequency. This effect would increase with increasing noise, i.e. decreasing CNR. Despite this possibly advantageous property of FBP_median_, transforming noise to signal, the distortion of the principle frequency to other frequencies was found to dominate.

The intention of using the non-negative constraint in the SIRT algorithm was not primarily to reduce noise^(28)^. Thus, the attenuation of air was set as the constraint level, and noise originating from CT numbers above air may not be directly reduced by this type of mechanism. However, it may reduce the convergence time^(28)^ and due to its mechanism, by cutting off the CT number at a certain level, it may help identify other nonlinear distortion effects such as those arising from metal artefacts. Metal artefacts may occur due to cut-offs at the maximum CT number of the CT system^(14)^. However, metal artefacts arise also from beam-hardening and/or photon starvation, which are other types of nonlinear effects creating distortion and requiring further study.

The results obtained with the SIRT_non-negative_ algorithm indicated an increase in the distortion, which is expressed as a fraction of the total output, when the CNR decreased. The reduction in distortion at higher frequencies may be due to a general loss in output signal as the noise increased. Thus, as the magnitude of the noise was reduced by the application of the non-negative constraint, the noise characteristics describing the origin of the signal contrast were lost. This may be due to a loss in signal resolution, which was observed in the PFR of the SIRT_non-negative_ algorithm. The increase in the DPS at lower values of CNR may be due to the frequency response to this nonlinear mechanism. PFR decreased through a narrowing of the curve, implying that the DC component is less sensitive to signal degradation. At the same time, the non-negative mechanism may have transformed the increased noise into signal distortion.

ΣDI provides a measure of the total distortion as a fraction of the total output signal. Thus, FBP_median_ appeared to generate the lowest total distortion among the algorithms investigated at CNR ≥ 0.5. However, ΣDI does not quantify the magnitude of the preserved signal. Hence, a low value of ΣDI may have been due to a general reduction in the total output signal. A comparison of ΣDI between the algorithms in the present study may then seem inadequate since the comparison will depend on the total output and the total output of FBP_median_ was lower than the other algorithms. However, this illustrates the necessity of comparing algorithms at the same output magnitude. Furthermore, the investigated FOM $\overline{\mathrm{DI}}$ reflects the mean of the DI, i.e. it quantifies the mean fraction of distortion independently of the total output.

In the present study, the nonlinear waveform distortion was quantified using sinusoidal task objects limited to the periodicity of a 32 × 32 pixel matrix. This method should be verified, for example, by increasing the number of task objects and by denser sampling of the principle frequencies. Also, the complexity of nonlinear distortion may lead to the use of only simple well-known task objects. Therefore, further investigations of the usefulness of these metrics in a more general context are necessary.

The magnitude of waveform distortion at sinusoidal principle frequencies was analysed in this study, but the frequency distribution of the distortion was not measured. An algorithm can cause artefacts that mimic anatomical or pathological structures, depending on the distribution of the distortion. Thus, other metrics characterising the distortion distribution should be developed.

## CONCLUSIONS

In the present work, the methodology involving the metrics PFR, DPS, DI and ΣDI was adapted for use in reconstruction algorithms for CT. Furthermore, a new FOM, $\overline{\mathrm{DI}}$, was proposed as a complement to ΣDI in comparisons between algorithms where the output signal differs substantially after reconstruction. The concept was tested through CT simulations by applying a nonlinear mechanism, a median filter and a non-negative constraint, to the reconstruction algorithms FBP and SIRT, respectively. Based on the results, it was concluded that the native CT reconstruction algorithms were not free from nonlinear waveform distortion. However, no CNR dependence was found in any of the metrics for these algorithms. Furthermore, the nonlinear mechanisms showed a clear and specific CNR dependence, indicating the necessity of distortion analysis in nonlinear CT reconstruction.

## FUNDING

This work was supported by grants from the Swedish state under the agreement between the Swedish government and the county councils, the Avtal om Läkarutbildning och Forskning agreement (ALFGBG-882281), the King Gustav V Jubilee Clinic Cancer Research Foundation (2017:154, 2018:225, 2019:270) and from the Department of Research and Development, NÄL (Norra älvsborgs länssjukhus), Uddevalla sjukhus, which are the two hospitals in the Hospital group
